# Deregulated molecules and pathways in the predisposition and dissemination of breast cancer cells to bone

**DOI:** 10.1016/j.csbj.2022.05.051

**Published:** 2022-05-30

**Authors:** Laijian Sui, Andrew Sanders, Wen G. Jiang, Lin Ye

**Affiliations:** a Cardiff China Medical Research Collaborative, Division of Cancer and Genetics, Cardiff University School of Medicine, Cardiff CF14 4XN, UK; b Joint surgery, Yantai Yuhuangding Hospital, Shandong 264000, PR China

**Keywords:** Breast cancer, Bone metastasis, Predisposition, Osteolytic and osteoblastic

## Abstract

**Background:**

Bone metastasis is the most common metastatic destination in advanced breast cancer, presenting a poor prognosis and clinical challenges in management. To date, the mechanism of bone metastasis in breast cancer remains largely unclear.

**Methods:**

Differentially expressed genes in primary tumours that developed bone metastases were systematically analysed using both TCGA-BRCA and E-MTAB-4003 databases. Adaptive phenotype in the subsequent bone lesions was analysed in the GSE46161 database. A series of biomarkers including homing, immune escape, angiogenesis, and factors involved in both osteoblastogenesis and osteoclastogenesis were included to dissect the molecular events underlying bone metastasis in breast cancer.

**Results:**

Upregulated expressions of GDF11 expression is positively correlated with colonization, osteoblastogenesis and osteoclastogenesis, whilst CD151 is positively associated with angiogenesis and immune escape. PAFAH1B2 expression is inversely correlated with the angiogenic process. Reduced YTHDF2 may facilitate cancer cell homing, osteoclastogenesis and immune escape in breast cancer. DPP9, FAS, ZNF519, RPP14 and FAU were evaluated for their potential involvement in for the homing to bone, escaping from immune surveillance, angiogenesis, osteoblastic activity and osteoclastic activity in the multi-step process of bone metastasis.

**Conclusion:**

GDF11, CD151, PAFAH1B2 and YTHDF2 may play a pivotal role in the predisposition of metastasis to the bone from breast cancer, whilst DPP9, FAS, ZNF519, RPP14 and FAU may be actively involved in the adaptative colonisation of metastatic breast cancer cells in bone.

## 1 Introduction

Cancer metastasis is a process which has gained increasing attention over the past decades. Bone is one of the most common metastatic sites, being frequently seen in certain solid tumours including lung, breast, prostate, colorectal, thyroid, gynaecologic, and melanoma. Over 70% of patients with advanced prostate or breast cancer patients present bone metastases [1]. Once cancer cells spread to the bone, it will gradually develop into a currently incurable disease and is associated with many symptoms including severe pain, pathological fracture caused by osteolysis, dysfunction of the limb, as well as hypercalcemia and bone marrow aplasia. In the early stages of bone metastasis, there are interactions within the bone microenvironment where the various stages of osteogenesis, osteolysis and haematopoiesis are systemic processed but spatially restricted. This intricately organised oncogenic process was first described in 1889 and was proposed by Stephen Paget [2]. The central rule of Paget’s hypothesis was the “soil” (bone microenvironment) and “seeds” (tumour cells) were compatible, with reciprocal actions amongst tumour cells, osteoblasts and osteoclasts lead to survival and colonization of cancer cells in the bone.

Previous reports have showed that bone is the most common metastatic site in breast cancer and approximately 80% of bone metastases from breast cancer presented destructive bone lesions characterised by osteolytic/osteoclastic metastasis [3], [4]. A panel of cytokines including interleukin 1 (IL1), interleukin 6 (IL6), parathyroid hormone like hormone (PTHrP) and colony-stimulating factor 1 (CSF-1) can induce the activation of osteoclasts in bone metastasis [5]. Similarly, a number of negative regulators of the osteolytic activity have been identified. For instance, DLC1-Rho signalling could block the PTHrP secretion induced by TGF-β (Transforming Growth Factor Beta), thereby suppressing the maturation of osteoclasts [6]. Receptor activator of nuclear factor Kappa-B ligand (RANKL) is crucial in modulating the differentiation and activity of osteoclasts. RANKL secreted by osteoblasts could specifically bind with RANK (receptor of RANKL) on osteoclast precursors to promote the differentiation of mature osteoclast [7]. N-telopeptide of type I collagens (NTX), a biomarker of bone resorption, is higher in osteoclastic disease. The ratio between urinary NTX and creatinine are now routinely monitored as a measure for bone resorption [8].

The molecular mechanism of osteoblastic lesions in breast cancer is still less characterised due to its much lower rates of presentation in the bone lesions from breast cancer. Recent studies have shown that core binding factor α1 (Cbfα1), also known as Runx-2, is closely related to osteoblastic differentiation [9]. Runx-2 was proved to have multiple roles being essential for the metastatic process [10]. Osteoblast cadherin (CDH11) was also shown to be an important stromal interaction protein in the osteoblastic metastasis in prostate cancer [11]. Other cytokines that enhance the growth, differentiation and activity of osteoblasts include platelet-derived growth factor (PDGF), fibroblast growth factor (FGF), TGF-β, bone morphogenetic protein (BMP) and Endothelin-1 [12]. Endothelin-1 can suppress the expression of the Dickkopf-1 (DKK-1) gene in bone marrow stromal cells [13]. When the inhibitory effect of DKK-1 for Wnt signalling is blocked, more active osteoblasts will be produced, which is conducive to the development of osteoblastic disease.

Despite good progress being made in the understanding of the molecular and cellular mechanisms of bone metastasis over the last two decades, clinical management and prognosis of bone metastasis remain unsatisfied. It is a profound challenge to unveil the pathology and vital molecules that are hijacked by metastatic cancer cells for their colonisation in bone. Bone metastasis is a complex process which consists of a series of interactions between cancer cells and the bone environment to achieve the successful homing, survival from immune surveillance and development of a secondary tumour with new vasculature in the bone. The present bioinformatic analysis was designed to screen key molecules involved in the bone metastasis of breast cancer and their possible role in the development of the disease.

## 2 Methods and database

### 2.1 Databases selection

RNA sequencing data of a cohort of breast cancer tumours (n = 1093) from The Cancer Gene Atlas (TCGA) was analysed as a cohort for discovery in the present study to evaluate differentially expressed genes (DEGs) in breast cancer with distant metastasis (n = 123), especially with bone metastasis (n = 58). Another breast cancer cohort, E-MTAB-4003, the gene array database comprising 720 tumours and 300 cases with bone metastasis was also employed in the current study as a cohort for validation. GSE464141 is a gene array database to provide the detailed characterization of breast cancer site-specific metastases, including bone metastasis, which was applied in the present study to identify the specially expressed genes associated with the bone metastasis adaptive phenotype.

### 2.2 Data collection and processing

The TCGA-BRCA cohort was applied as the discovery cohort to identify the DEGs in breast cancer with distant metastasis and the bone metastasis developed subsequently. Then,DEGs were validated in the E-MTAB-4003 dataset to identify the bone metastasis predeposit genes. Correlation of DEGs with osteolytic and osteoblastic markers was analysed in the E-MTAB-4003 cohort. The TCGA-BRCA cohort was further used to explore the altered expression of DEGs with bone metastasis survival analysis. The association between the DEGs and osteoclastic factors was further evaluated in TCGA-BRCA dataset using Spearman tests.

Following the analysis of the genes associated with the bone metastasis disposition, the adapting phenotype of cancer cells in bone niches was explored. Genes with significantly differential expression between primary tumours and tumours subsequently developed bone metastasis were analysed in gene array corhort (GSE46141). On-going analysis was performed to investigate the adaptive phenotype in immune surveillance escape and neovascular in the bone site.

### 2.3 Statistical analysis

Normally distributed data was analysed by student T-test, whilst non-normally distributed data was assessed using Mann-Whitney tests. Bone metastasis free survival was assessed using Kaplan-Meier tests in TCGA-BRCA database. Correlation with markers of homing, osteoclastogenesis, osteoblastogenesis, immune-escape and neo-angiogenesis was evaluated using Spearman test. P < 0.05 was regarded as a statistically significant difference. T-test, Mann-Whitney test, Spearman correlation test and Kaplan-Meier survival analysis were performed using SPSS 27.0 (IBM UK Ltd, Bristol, UK). The GraphPad Prism v.9.0 (GraphPad Systems Inc.) program was applied to analyse the data.

## 3 Results

### 3.1 Key molecules/pathways associated with predisposition of cancer cells spreading to bone.

**Fig. 1:**
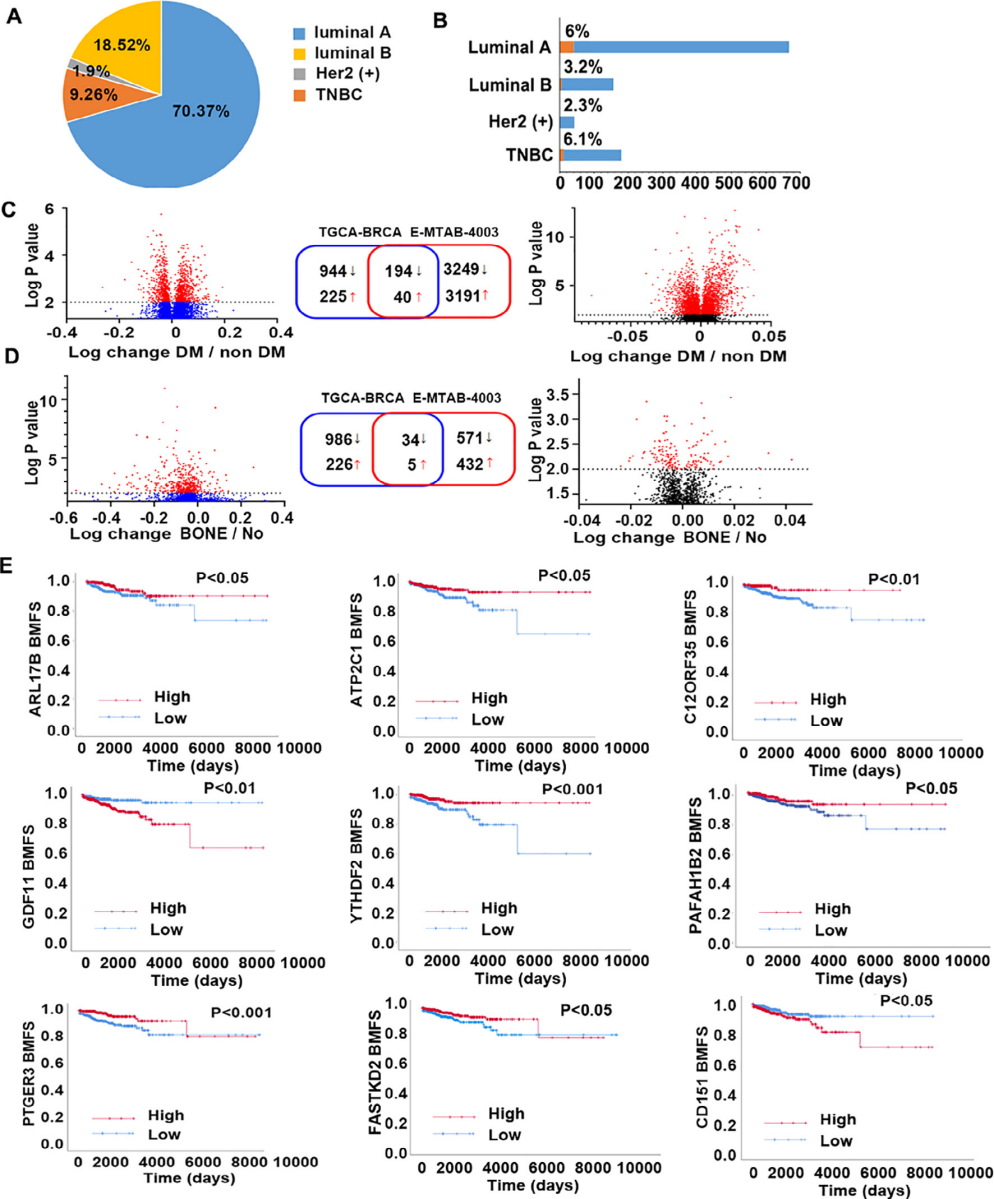
Differentially expressed genes in breast cancer and bone metastasis. (A) Pie chart shows percental distribution of different subtypes of breast cancer analysed in TCGA-BRCA database. (B) Incidence of bone metastasis in four breast cancer subtypes. (C) Differential expressed genes associated with distant metastasis in both TCGA-BRCA (n = 1093) and E-MTAB-4003 cohort (n = 720). (D) Bone metastasis specific genes in TCGA-BRCA and E-MTAB-4003 database. (E) Kaplan-Meier survival analyses in TCGA-BRCA database showed associations between the bone metastasis related DEGs and bone metastasis free survival.

Initial analyses were performed in the TCGA-BRCA cohort to identify deregulated genes that were associated with distant metastases. This led to a discovery of 1138 genes that were decreased significantly in primary tumours which developed distant metastases when comparing with the cases without distant metastasis. Of the 1138 genes 194 were also reduced in the primary tumour with distant metastases in the E-MTAB-4003 cohort in which 3343 genes were lowly expressed in those primary tumours with distant metastases (Fig. 1C). On the other hand, 40 genes were markedly increased in the primary tumours which developed distant metastases in both TCGA-BRCA and E-MTAB-4003 cohorts (Fig. 1C).

DEGs associated with bone metastasis were identified in the primary tumours which had bone metastases in comparison with the primary tumours without distant metastasis. The bone metastasis related DEGs were likely to relate with the predisposition of spread to bone. To identify genes specifically associated with bone metastasis, a further analysis was performed in comparison with their expression in the primary tumours which developed other distant metastases except the bone. Thirty-nine genes were identified as candidate genes for the predisposition in both cohorts, in which 34 genes are reduced and 5 genes are upregulated (Fig. 1D). The five upregulated genes are GPR20 (G-Protein Coupled Receptor 20), GLCE (Glucuronic Acid Epimerase), CD151, ACTL8 (Actin Like 8) and GDF11. Furthermore, 9 of the 39 genes exhibit significant association with bone metastasis free survival in the TCGA-BRCA cohort (Fig. 1E).

Among these genes, upregulated expressions of GDF11 (Growth Differentiation Factor 11) and CD151 were associated with bone metastasis, whilst lower expressions of ARL17B (ADP-Ribosylation Factor-Like Protein 17), ATP2C1 (ATPase Secretory Pathway Ca2 + Transporting 1), C12orf35 (also named RESF1, Retroelement Silencing Factor 1 ), YTHDF2 (YTH N6-Methyladenosine RNA Binding Protein 2), PAFAH1B2 (Platelet Activating Factor Acetylhydrolase 1b Catalytic Subunit 2), PTGER3 (Prostaglandin E Receptor 3) and FASTKD2 (FAST Kinase Domains 2) are associated with higher risk of bone metastasis (Fig. 1E).

### 3.2 Survival and evasion from immune reaction in bone marrow.

Evasion from immune attack is vital for disseminating cancer cells to survive on their arrival in the bone and subsequent colonisation. According to the previous study, possible immune-escape mechanism between disseminated cells and immune cells are summarised as follows. Disseminated cells secret LDH5 (Lactate Dehydrogenase 5) and ADAM10 (A Disintegrin and Metalloproteinase Domain 10) to survive from being recognised and diminished by NK-cells. ADAM10 contributes to the deregulation or shedding of MICA (MHC Class I Polypeptide-Related Sequence A) and MICB (MHC Class I Polypeptide-Related Sequence B) (ligand of NKG2D). LDH5 increases the expression of NKG2D ligands on monocytes, which leads to the downregulation of NKG2D in NK cells. Cytokeratins, such as CK8, CD18 and CK19, can intercept the recognition of T cell receptors. Downregulated MCH I expression is also important in escaping from immune surveillance. Expression of PD-L1 (the immune-checkpoint inhibit protein), CD47 (key molecule expressing ‘do not eat me signal’) and apoptotic protein FAS (also named TNFRSF6, Tumour Necrosis Factor Receptor Superfamily Member 6) are all crucial in participating the immune-escape mechanism of tumour cells. The Irf7 (Interferon Regulatory Factor 7) pathway exerts a powerful role in suppressing the onset of bone metastases of breast cancer (well-reviewed in [14]).

**Fig. 2:**
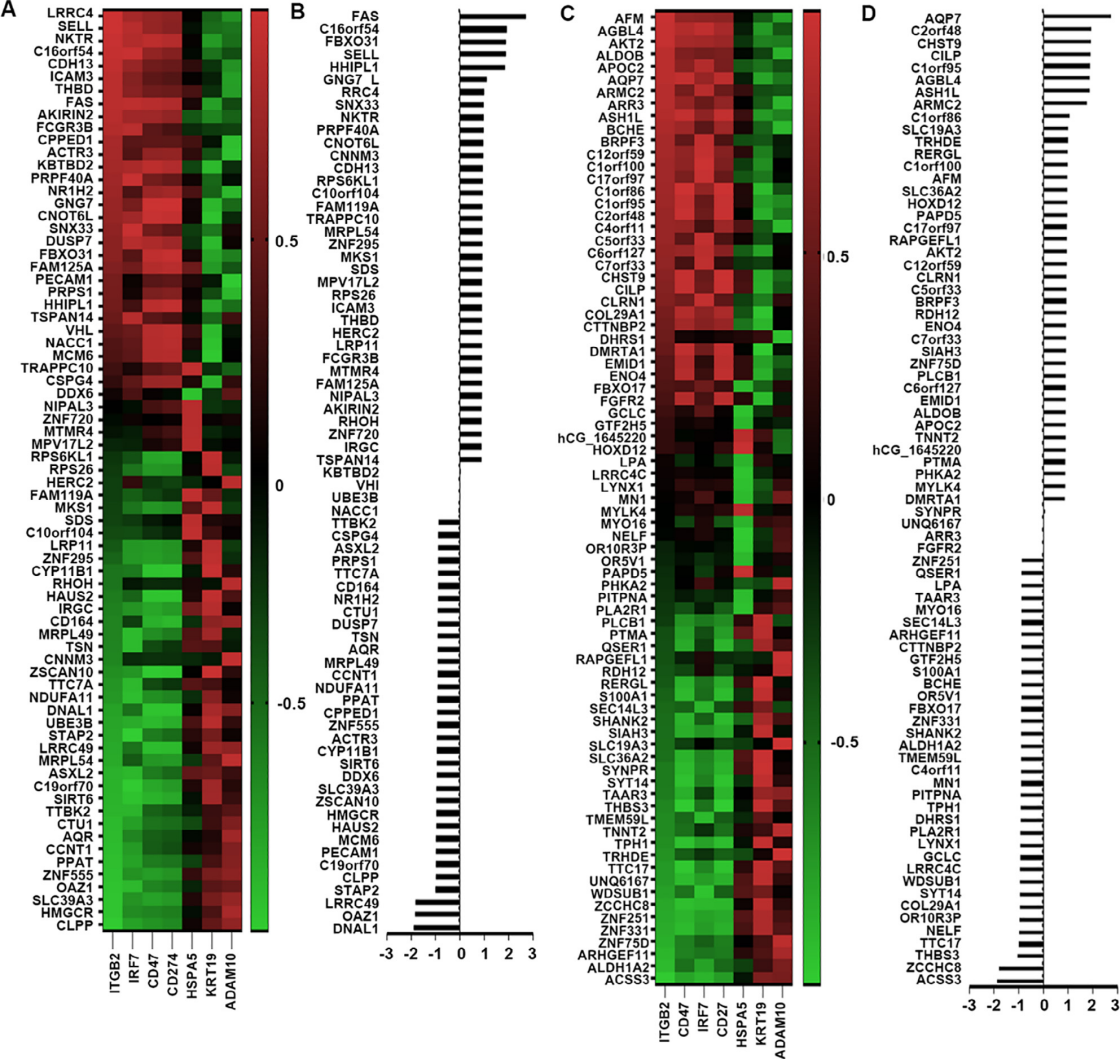
DEGs associated with immune escape in bone lesion. Heatmap to show the upregulated (Fig. A) and the downregulated (Fig. C) genes correlated with escaping from the immune surveillance. Statistical correlation scores of the upregulated and downregulated genes were summed and presented in histogram (B) and (D), respectively.

In the present analyses, CD18 (ITGB2), IRF7, CD47, PDL1(CD274), HSPA5 (Heat Shock Protein Family A (Hsp70) Member 5), CK19 (KRT19, Keratin 19) and ADAM10 were applied as the biomarkers to assess the specific genes involved in the immunal-surveillance escape (Fig. 2 A and C). The correlation efficiencies were summed to investigate the role of these genes (Fig. 2 C and D). 37 genes displaying upregulated expression in the bone metastatic lesion presented positive correlation with these biomarkers, whilst another 37 genes with downregulated expression profiles presented inverse correlation with these biomarkers. FAS, C16orf54, FBXO31 (F-Box Only Protein 31), SELL (Selectin L), HHIPL1 (HHIP-Like Protein 1) and GNG7 (Guanine Nucleotide-Binding Protein G(I)/G(S)/G(O) Subunit Gamma-7) were the top six genes positively associated with the Survival and evasion from immune reaction in bone marrow. Meanwhile, ACSS3 (Acyl-CoA Synthetase Short Chain Family Member 3), ZCCHC8 (Zinc Finger CCHC Domain-Containing Protein 8), THBS3 (Thrombospondin 3), TTC17 (Tetratricopeptide Repeat Domain 17), NELF (Negative Elongation Factor) and OR10R3P (Olfactory Receptor Family 10 Subfamily R Member 3 Pseudogene) were also closely correlated with the immuno-surveillance escape, presenting reduced expression.

### 3.3 Adapting phenotype of metastatic cancer cells in bone niches

**Fig. 3:**
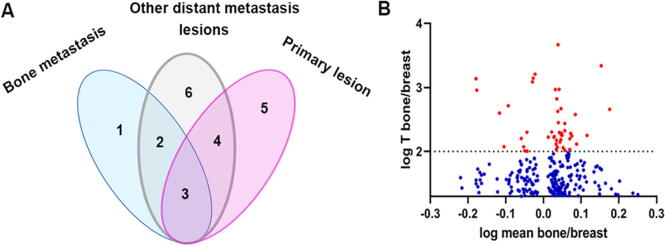
Molecules and signalling pathways involved in the adaptive phenotype in bone niches. (A) Peddle plot to present how specific genes correlated adaptive phenotype in bone metastasis were selected. (B) Genes correlated with adaptive phenotype in bone metastasis were showed in volcano plot.

DEGs associated with adaptive phenotype in the bone niche were identified by analysing genes expressed in bone metastatic lesions, primary breast cancer and other distant metastases in the GSE46161 database. 211 increased genes, and 217 decreased genes were regarded as differentially expressed genes contributing to the adaptation in bone niches (Section 1 in Fig. 3A).

**Fig. 4:**
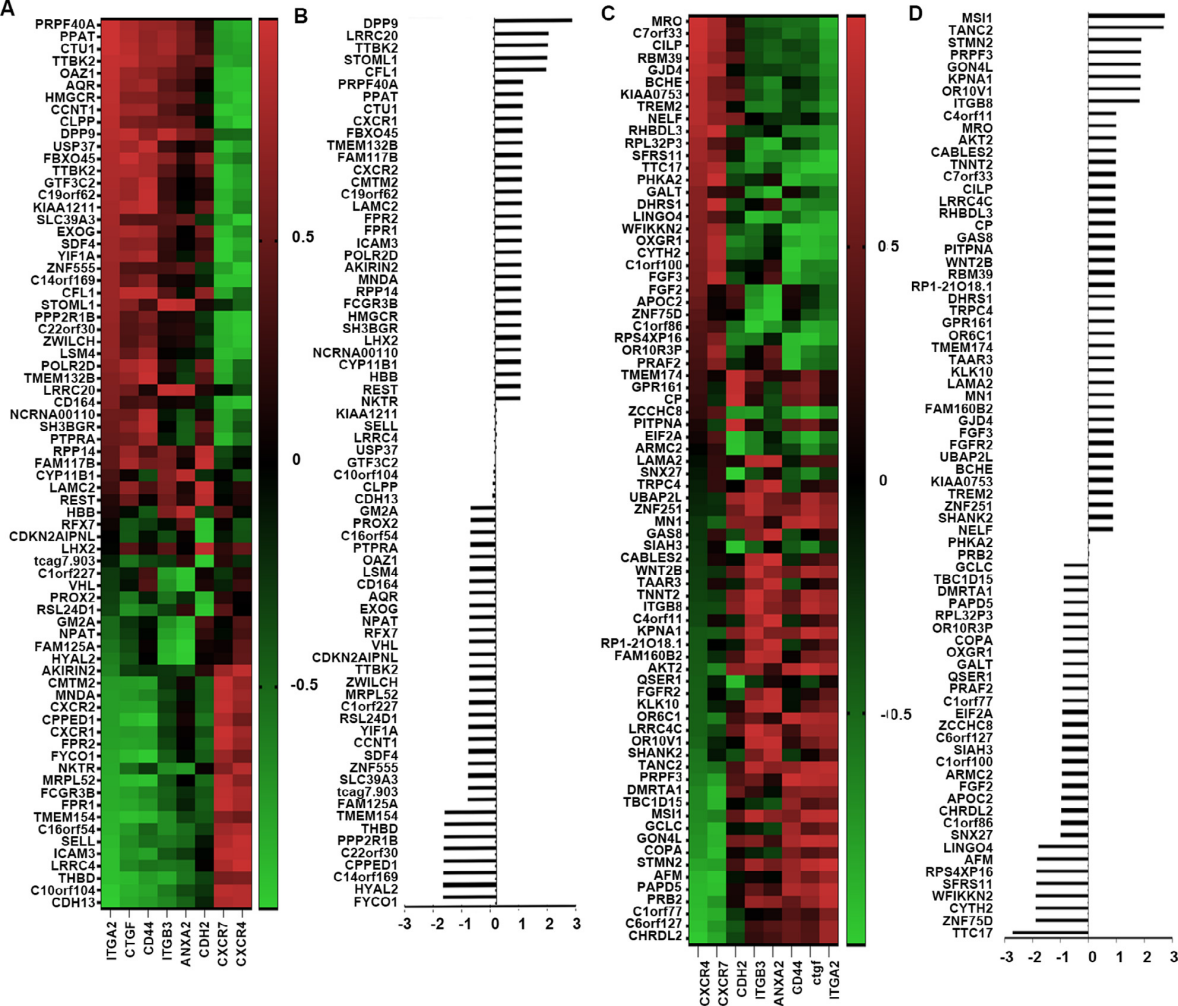
Adaptive DEGs in bone metastases and bone homing markers. (A) Correlation between the upregulated adaptive DEGs and bone homing markers. (B) Histogram shows sum of correlation coefficients with the homing factors. (C) Correlation between the downregulated adaptive DEGs and the homing markers. (D) Sum of correlation coefficients with the homing factors for each downregulated adaptive DEGs are presented in the histogram.

According to the previous research, eight genes including CXCR4 (C-X-C Motif Chemokine Receptor 4) [15], CXCR7 (C-X-C Motif Chemokine Receptor 7) [16], CDH2 (Cadherin 2) [17], ITGB3 [18], ANXA2 [19], CD44 [9], CTGF [9] and ITGA2 [18] were proved to exert pivotal role in mediating the colonization of the disseminated tumours, which were applied to investigate the correlation between DEGs and the adhesion. 155 genes presented high correlation with these biomarkers. From our analysis, 45 genes out of 234 increased DEGs presented high positive correlation with these adhesive markers, whilst 35 genes were negatively correlated with these biomarkers (Fig. 4 A and B). For the decreased DEGs, 38 genes were found positively associated with adhesive markers, another 37 genes showed negative correlation with adhesion markers. MSI1 (Musashi RNA Binding Protein 1), TANC2 (Tetratricopeptide Repeat, Ankyrin Repeat And Coiled-Coil Containing 2), STMN2 (Stathmin 2), PRPF3 (Pre-MRNA Processing Factor 3) and GON4L (Gon-4 Like) were the top five molecules positively correlated with the adhesive markers with increased expression in the bone lesions, whilst FYCO1 (FYVE And Coiled-Coil Domain-Containing Protein 1), HYAL2 (Hyaluronidase 2), C14orF169 (also named RIO1, Ribosomal Oxygenase 1), CPPED1 (Calcineurin Like Phosphoesterase Domain Containing 1) and C22orf30 (Chromosome 22 Open Reading Frame 30) were the top five genes negatively associated with these markers (Fig. 4 B and D). As the top selected gene associated with the homing of the tumour cells in the bone environment, DDP9 (Dipeptidyl Peptidase 9) expression was highly positively with ITGA2 (Integrin Subunit Alpha 2) (r = 0.885, P < 0.05), CTGF (also called CCN2, Cellular Communication Network Factor 2) (r = 0.926, P < 0.05) and ITGB3 (Integrin Subunit Beta 3) (r = 0.917, P < 0.05). TTC17 (Tetratricopeptide Repeat Domain 17), as one of the downregulated genes was the leading gene inversely correlated with homing markers. It was also inversely correlated with ITGB3 (r = -0.879, P < 0.05), CTGFA2 (r = -0.945, P < 0.05), and ITGA2 (r = -0.893, P < 0.05).

### 3.4 Tumour associated angiogenesis in bone metastasis

Both the bone and tumour need vasculature; in fact, a proper bone turnover is coupled with angiogenesis and recruitment of blood vessels [20], while tumour-induced neo-angiogenesis is a key event and a hallmark of tumour progression and aggressiveness. The Vascular Endothelial Growth Factor (VEGF)/VEGF receptor (VEGFR) axis is the most prominent pathway both in physiological and tumour-induced neo-angiogenesis and is a therapeutic target in different types of cancers [21]. Besides VEGF, Fibroblast growth factor 2 (FGF2), platelet derived growth factor (PDGF), angiopoietins (ANGPTs) and Eph/ephrin signalling etc. were also identified as the pivotal pro-antigenic factors. [22].

**Fig. 5:**
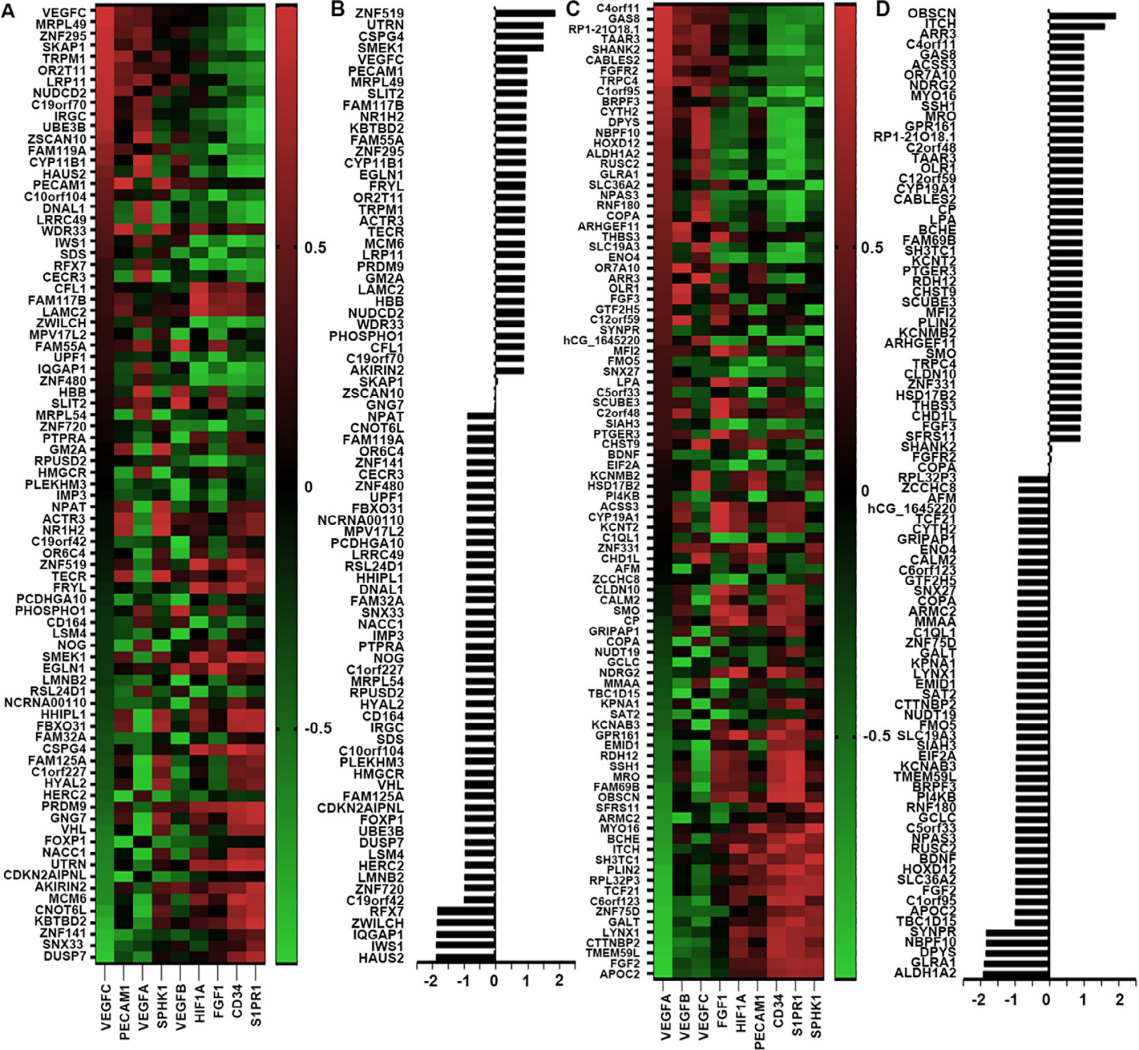
Aberrantly expressed genes correlating with neo-angiogenesis in the bone lesion. Heatmap to show the upregulated (Fig. A) and the downregulated (Fig. C) genes correlated with tumour angiogenesis. Statistical correlation co-efficiency of the upregulated and downregulated genes was summed and presented in histogram (B) and (D), respectively.

From our analyses, 33 genes with upregulated expression presented a positive association with the angiogenesis markers (Fig. 5B), whilst 55 downregulated genes were negatively correlated with the angiogenesis markers (Fig. 5D). Top 5 genes with increased expression involved in the angiogenesis were ZNF519 (Zinc Finger Protein 519), UTRN (Utrophin), CSPG4 (Chondroitin Sulfate Proteoglycan 4), SMEK1 (also named PPP4R3A, Protein Phosphatase 4 Regulatory Subunit 3A) and VEGFC, while ALDH1A2, GLRA1 (Glycine Receptor Alpha 1), DPYS (Dihydropyrimidinase), NBPF10 (Neuroblastoma Breakpoint Family Member 10) and SYNPR (Synaptoporin) were the top 5 downregulated genes associated with the tumour angiogenesis.

### 3.5 Involvement of differential expressed genes in the progression of bone metastasis.

**Fig. 6:**
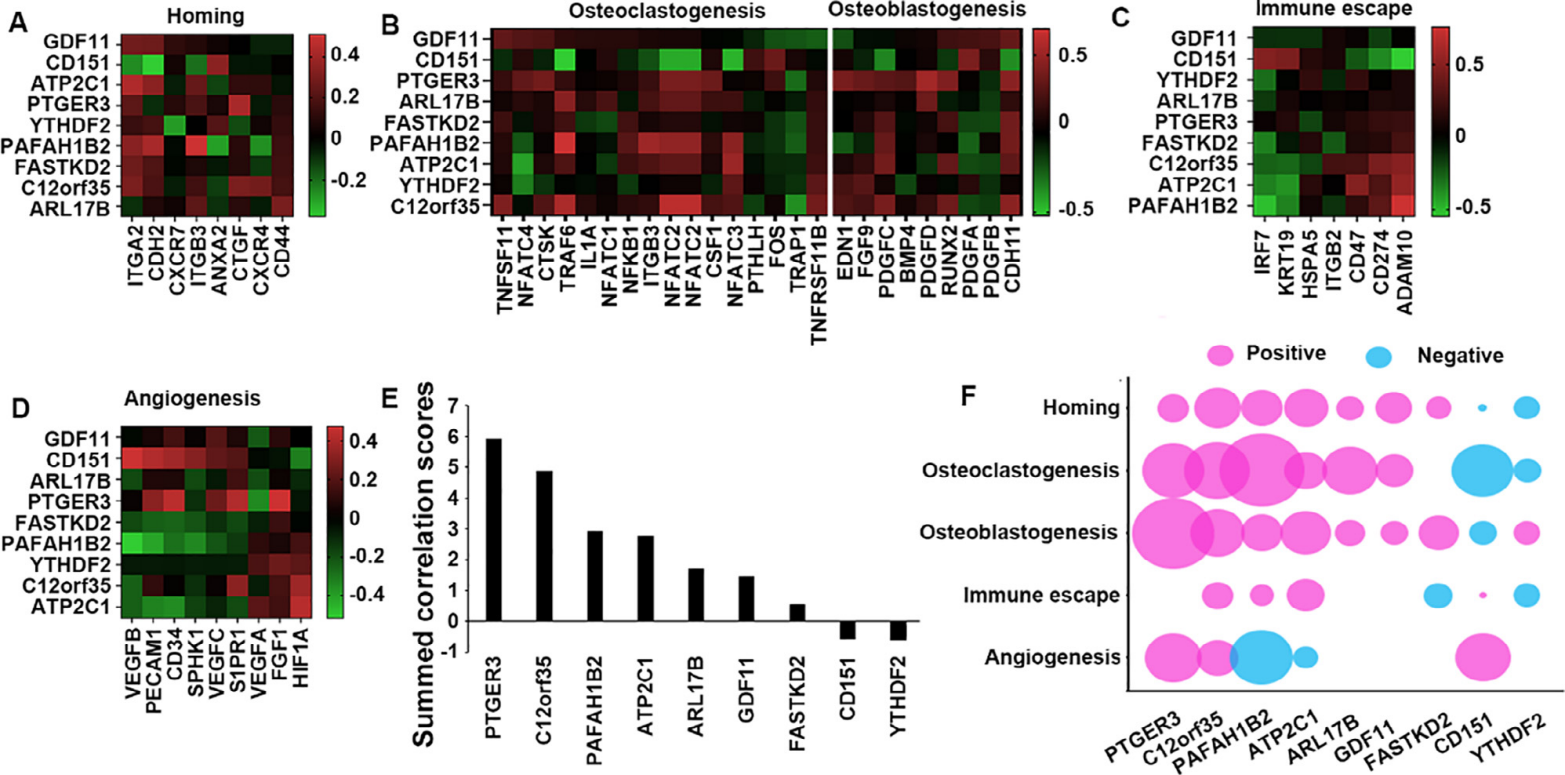
Role of predispositivon related factors in the bone metastasis. Heatmap to present the correlation between bone metastasis predisposive genes and homing (A), osteoclastogenesis, osteoblastogenesis (B), immuno-escape (C), angiogenesis (D) markers. (E) Correlation co-efficiency was summed up and presented as histogram. (F) Bubble plot exhibitng overall correlation between these 9 predisposition related genes and 5 key bone metastatic events.

Involvement of the bone metastasis was further assessed for the nine identified genes by a series of index consisting by bone metastasis markers including homing (Fig. 6A), osteoclastogenesis regulators (Fig. 6B), osteoblastogenesis regulators (Fig. 6E), immune escape (Fig. 6C), and tumour angiogenesis (Fig. 6D) in the TCGA-BRCA database. From the analyses, GDF11 was positively correlated with the homing markers (ITGA2 and CDH2, r = 0.278, P<0.05 and r = 0.268, P<0.05), osteoclastic markers (NFATC4 (Nuclear Factor of Activated T Cells 4) and RANKL (Receptor Activator of Nuclear Factor Kappa-B Ligand), r = 0.269, P<0.05 and r = 0.311, P<0.05 respectively), osteoblastic marker (CDH11 r = 0.323, P<0.05). Upregulated CD151 expression was positively correlated four angiogenesis markers, VEGFB (r = 0.471, P < 0.01), PECAM1 (Platelet and Endothelial Cell Adhesion Molecule 1) (r = 0.410, P < 0.01), CD34 (r = 0.374, P < 0.01) and SPHK1 (Sphingosine Kinase 1) (r = 0.306, P < 0.05). CD151 was also positive correlated with ANXA2 (Annexin A2) (homing marker r = 0.344, P<0.01), FOS (osteoclastic regulator r = 0.345, P<0.01), PDGFB (Platelet Derived Growth Factor Subunit B) (osteoblastic regulator r = 0.395, P < 0.05)), IRF7 (immune escape marker r = 0.483, P<0.01), KRT19 (Keratin 19) (immune escape marker r = 0.451, P<0.01). For the downregulated genes, YTHDF2 (YTH N6-Methyladenosine RNA Binding Protein 2) expression was negatively correlated with the homing marker (CXCR7(r = -0.284, P < 0.05)), the osteoclastic regulator (NFATC4 (Nuclear Factor of Activated T Cells 4) (r = -0.323, P < 0.05)) and immune escape marker, IRF7 (r = -0.293, P < 0.05). PAFAH1B2 expression was highly inversely correlated with angiogenesis markers, for instance VEGFB (r = -0.515, P < 0.01), PECAM1 (r = -0.415, P < 0.01), CD34 (r = -0.285, P < 0.05) and SPHK1 (r = -0.342, P < 0.01). C12orf35, ATP2C1 (ATPase Secretory Pathway Ca2 + Transporting 1), ARL17B (ADP Ribosylation Factor Like GTPase 17B), FASTKD2 and PTGER3 (Prostaglandin E Receptor 3) were also inversely associated with various molecules involved in bone metastasis but these did not reach a statistically significant level. Overall correlation with these bone metastasis related markers was analysed by a sum of correlation coefficients for the 9 predisposition genes individually which is shown as a histogram (Fig. 6E). A bubble plot was used to show their influence on each aspect of bone metastasis, respectively (Fig. 6F).

**Fig. 7:**
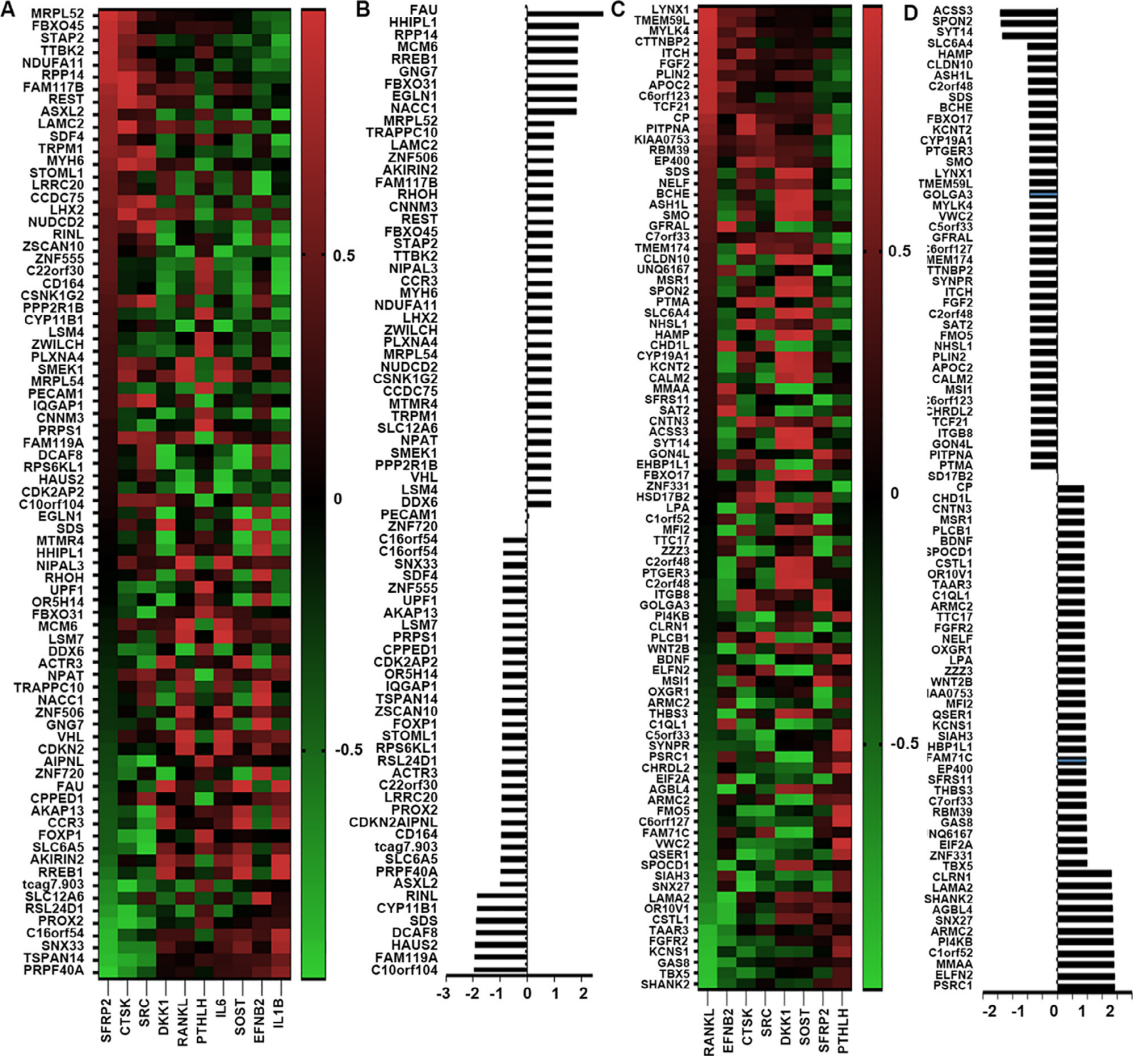
Determining factors associated with osteoclastic bone lesions. (A) Heatmap to show the increased genes correlated with osteoclastic markers. (B) Statistical correlation scores of upregulated genes were summed and presented in histogram. (C) Heatmap to show the decreased genes correlated with osteoclastic markers. (D) Statistical correlation scores of downregulated genes were summed and presented in histogram.

Generally, breast cancer bone metastasis manifests as osteolytic bone metastasis. A series of molecules including RANKL, OPG (Osteoprotegerin), Cathepsin-K (CTSK), SRC (SRC Proto-Oncogene, Non-Receptor Tyrosine Kinase), IL-6 etc. were used as the osteoclastic signature to screen possible vital genes in osteoclastogenesis. Statistical correlation scores with the osteoclastic regulatory genes were then summed to assess the influence of genes in the osteoclastogenesis. 42 upregulated genes with positive correlation (Fig, 7A and B) and 41 downregulated genes negatively correlated with osteoclastic markers were selected out from all the aberrant expressed genes (Fig. 7C and D). From this assessment, increased FAU, HHIPL1, RPP14 (Ribonuclease P/MRP Subunit P14) with positive correlation and decreased ACSS3, SPON2 (Spondin 2), SYT14 (Synaptotagmin 14) with highly inverse association were identified as potential molecules with osteoclastogenesis (Fig. 7D). However, there were also several genes exhibited negative association with the osteoclastogenesis, such as ACSS3, SPON2, SYT14 etc. They were highly positively correlated with osteoclastic markers but presented decreased expression. There were also a series of upregulated genes exhibited highly negative correlation with selected osteoclastic markers, such as C10orf104, FAM119A, HAUS2 (HAUS Augmin Like Complex Subunit 2) etc. (Fig. 7B).

**Fig. 8:**
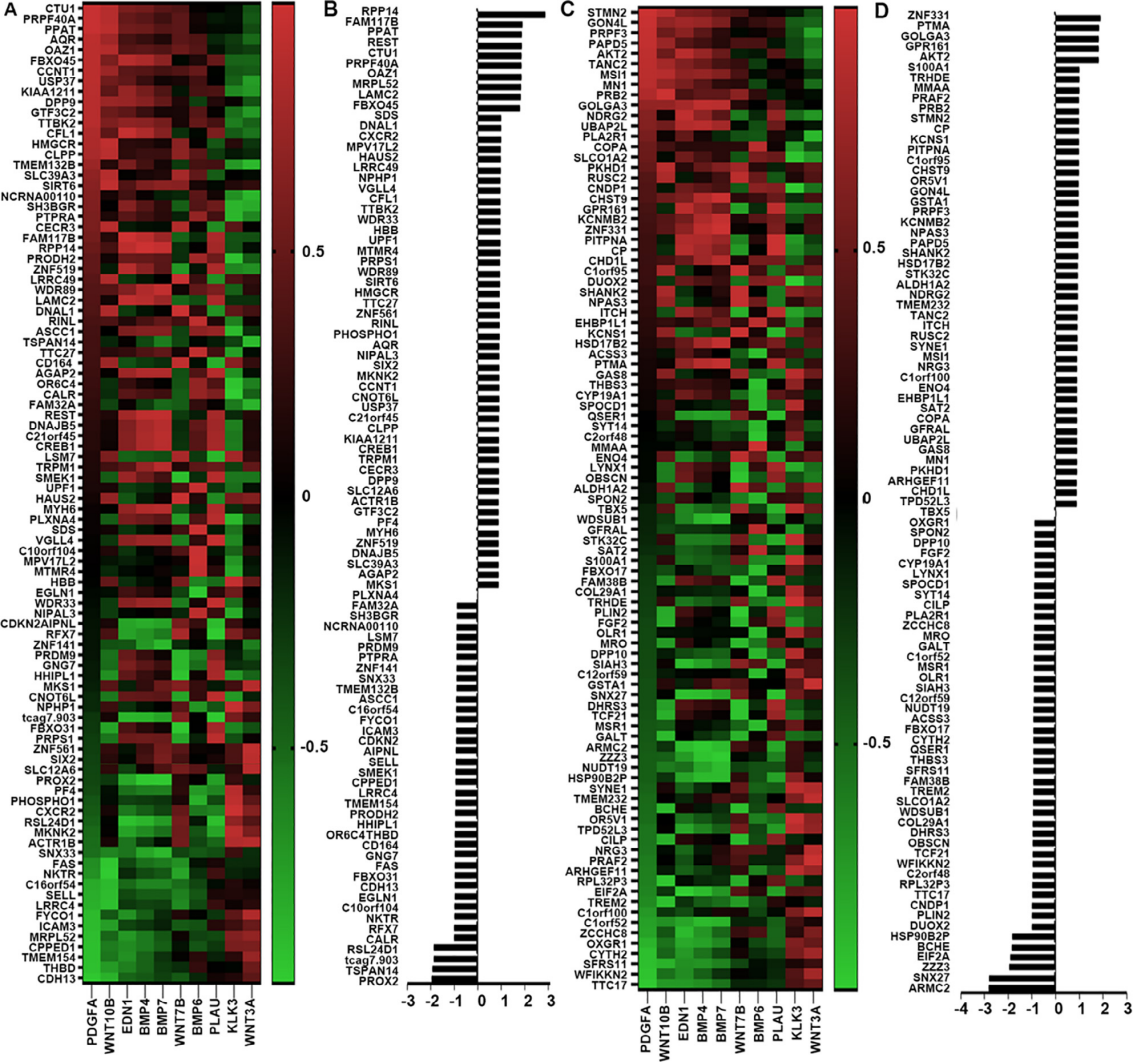
Determining factors associated with osteoblastic bone lesions. Heatmap to show the increased and decreased genes correlated with osteoblastic markers respectively (A and C). Statistical correlation scores of increased and decreased genes were summed and presented in histogram. (B and D).

As another important pattern of bone metastasis, osteoblastic metastasis is mainly seen in prostate cancers. However, osteoblastic bone metastasis also exists with the osteoclastic bone metastasis simultaneously in the breast cancer. in the GSE46161 cohort, 56 upregulated genes were positively associated with osteoblastic markers from our assessment, whilst 46 downregulated genes were negatively correlated with osteoblastic biomarkers. These 102 genes were preliminary regarded as the osteoblastic bone metastasis promoters. Among them, upregulated RPP14 (Ribonuclease P Protein Subunit P14), FAM117B (Family With Sequence Similarity 117 Member B), REST (RE1 Silencing Transcription Factor), CTU1 (Cytosolic Thiouridylase Subunit 1), PRPF40A (Pre-MRNA Processing Factor 40 Homolog A), OAZ1 (Ornithine Decarboxylase Antizyme 1) (Fig. 8B) and downregulated ARMC2 (Armadillo Repeat Containing 2), SNX27 (Sorting Nexin 27), ZZZ3 (Zinc Finger ZZ-Type Containing 3), EIF2A (Eukaryotic Translation Initiation Factor 2A), BCHE (Butyrylcholinesterase), HSP90B2P (Heat Shock Protein 90 Beta Family Member 2, Pseudogene) presented high correlation scores. (Fig. 8D) As the top three molecules associated with the osteoblastic bone metastasis from the current analyses, RPP14 was highly positively correlated with BMP4 (r = 0.999, P < 0.01), BNMP7 (r = 0.954, P < 0.05), EDN1 (Endothelin 1) (r = 0.913, P < 0.05), whilst SNX27 was inversely associated with BMP4 (r = -0.927, P < 0.05), BMP7 (r = -0.939, P < 0.05), PLAU (Plasminogen Activator, Urokinase) (r = -0.941, P < 0.05).

## 4 Discussion

As the most preferential distant metastasis site in breast cancer, bone metastasis is a multi-step cascade which contains several key steps: local invasion, dissociation from primary tumour, intravasation, spread through blood circulation, extravasation, and colonisation in bone [23], [24]. Acquiring capabilities to disseminate is the initial and indispensable step for cancer cells to advance from a primary to a metastatic tumour. From our analyses, 134 genes presented differential expression in the primary lesions with distant metastases, compared with those without, in both TCGA-BRCA and E-MTAB-4003 datasets. Further assessment was performed to identify differentially expressed genes in the primary tumours that spread to bone specifically. Aberrantly expressed genes in the primary tumours with bone metastasis were compared with those without distant metastasis. Subsequently, the genes associated with distant metastasis were excluded from the genes differentially expressed in the bone metastasis. The remaining molecules were regarded as genes associated with bone metastasis. Following this, bone metastasis free survival analysis was performed to further evaluate the candidate factors associated with the predisposition. Nine genes from 39 molecules presented significant alterations bone metastasis free survival with altered expressions. Upregulated GDF11 and CD151 expressions were positively correlated with bone metastasis, whilst the inverse correlation was shown with the other seven. Subsequent analyses on the impact of these promising genes involved in bone metastasis showed that GDF11 may involve in the homing, osteoclastogenesis and osteoblastogenesis in bone colonization. GDF11 was demonstrated to reduce the bone mass through promoting the RANKL induced osteoclastogenesis and suppressing the osteoblastogenesis in mice via Smad 2/3 (Mothers Against Decapentaplegic Homolog 2,3) and Nfatc1 (Nuclear Factor Of Activated T Cells 1) induced by C-FOS [25]. GDF11 inhibitor has been shown to reduce the bone loss in mouse model [26]. However, the proposed involvement of GDF11 in bone metastasis is yet to be investigated to shed light on its predictive and therapeutic potential. YTHDF2 was found to participate in Smad-dependent signalling to inhibit the differentiation of osteoblasts via stablizing Smad7 and Smurf1 (SMAD Specific E3 Ubiquitin Protein Ligase 1), which suppresses the phosphorylation of Smad1/5/9, then expression of target genes such as Runx2, Col1a1 (Collagen Type I Alpha 1 Chain), ALP (Alkaline Phosphatase) etc. were downregulated, leading to the reduced osteogenesis [27]. The present analyses showed that YTHDF2 was negatively correlated with the homing marker (CXCR7) and immune escape marker (IRF7), which indicates that YTHDF2 may also involve in the homing and immune escape mechanism. CD151, known as a member of the tetraspanin family, is actively involved in cancer progression via binding integrins and regulating growth factor receptors. CD151 has been shown to mediate communication between PC3 prostate cancer cells and the bone environment and promoted the migration and invasion of the tumours. CD151 knockdown in PC3 cells inhibited the activation of pro-migration kinases mediated by osteoblasts [28]. According to Zhang’s study, CD151 promoted migration in osteosarcoma through upregulating the transcripts of matrix metalloproteinase 9 (MMP9) via glycogen synthase kinase3 (GSK-3β)/β-catenin signalling pathway [29]. From our analyses, CD151 is mainly correlated with the angiogenesis markers in the bone lesion, which indicated that CD151 may act as a pivotal molecule mediating the neo-vascularisation in the subsequent lesions.

**Fig. 9:**
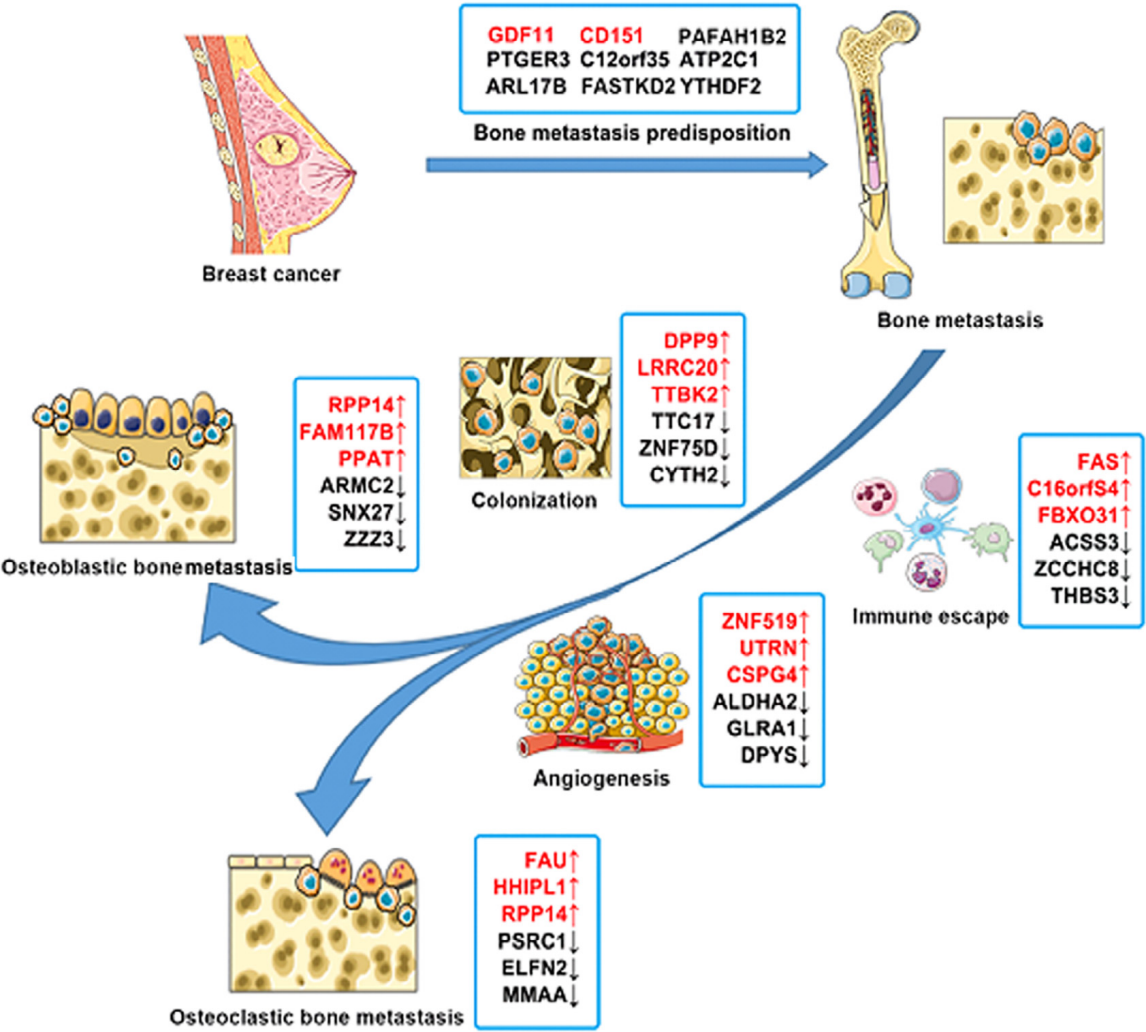
Genes involved in the predisposition and adaptive phenotype of bone metastasis in breast cancer were summarised and illustrated with Servier Medical Art tools (http://servier.com).

Bone colonization occurs more than the passive circulated spread, which is preferentially initiated from the adhesion to bone marrow endothelium *in vitro* study [30], [31]. Due to the indispensable role in bone reconstruction and the formation of hematopoietic stem cell niche, bone marrow endothelium has been shown to promote tumour cells to populate the trabecular bone. A series of adhesion factors excreted by bone marrow endothelia such as selectins [32], [33] and galectin-3 [34] were not only the vital mediator for hematopoietic stem cell (HSC) homing, they also actively participated in tumour cells usurping the bone. Research in interrupting the adhesion between tumour and endothelial cells found it significantly decreased the occurrence of bone metastasis [35]. Chemotaxis, especially for the CXC chemokine receptor-4 (CXCR4) was proved to exert a pivotal role in regulating the metastasis of breast cancer to bone [36]. From our analyses, DPP9 (Dipeptidyl Peptidase 9), LRRC20 and TTBK2 expressions were the top three upregulated genes that may be associated with the homing of the migrated tumour cells in the bone. DPP9 is a kind of metalloproteases, belonging to the dipeptidyl peptidase (DPP) family. DPP9 was found to regulate cell behaviour via the epidermal growth factor (EGF) signalling pathway [37]. DPP9 knockdown in Ewing sarcoma induced cell death through a regulation of cytokines such as poly, polymerase-1 and apoptosis-inducing factor [38]. Little research was reported on its involvement in breast cancer. Previous bioinformatics revealed the upregulated DPP9 expression in breast cancer was associated good prognosis. In the current analyses, DPP9 expression was highly correlated with homing markers, for example ITGA2 and CTGF, which indicated that DPP9 may also be involved in the homing of disseminating cancer cells in the bone via degrading the metalloproteins to facilitate the migrated cells adherence to the bone matrix. Notably, DPP9 prefers to regulate the cell behaviour through EGFR signalling which is a dominant signalling in the TNBC subtype of breast cancer, DPP9 tends to be a pivotal molecule in TNBC. However, the exact role of DDP9 in the colonisation of breast cancer cells in bone, particularly the TNBC cells and preventive potential of targeting DDP9 are yet to be explored. LRRC20 was reported to correlate with disease manifestations and severity of systemic lupus erythematosus (SLE) [39]. From our analyses, LRRC20 was positively correlated with ITGB3 and ANXA2. Beside from facilitating cell colonization, ANXA2 also actively heightened osteoclast formation and bone resorption, which indicates that LRRC20 may also participate in the osteoclastogenesis in the bone metastasis process. TTBK2 was found to modulate the cell proliferation and invasion in glioma cell lines via miR-520b/EZH2 axis [40]. Attenuated TTBK2 expression inhibited the proliferation, migration and invasion of Glioma cells via modulating miR-1283 and CHD1 [41]. The present study shows that TTBK2 expression is highly correlated with ITGA2 and CTGF which enhance cell adhesion. It suggests that TTBK2 may promote the homing of metastatic cancer cells in the bone. CTGF plays a vital role in chondrocyte proliferation, combined with the previous study on the role of TTBK2 in regulating the cell proliferation, together with ITGA2 and CTGF, TTBK2 may coordinate the adhesion and proliferation of the migrated breast cancer cells in the bone subsequent lesion. TTC17, ZNF75D and CYTH2 were the top three downregulated genes inversely correlated with usurp the bone in the current analyses (Fig. 9). TTC17 was reported to involve in the polymerization of actin and ciliogenesis [42]. Circ-TTC17 was found to enhance the progression of esophageal squamous cell carcinoma by promoting proliferation and migration [43]. It suggests that upregulated DPP9, LRRC20 and TTBK2 together with downregulated TTC17, ZNF75D and CYTH2 may play pivotal role in the orchestrated adaptive homing of metastatic breast cancer cells in bone niche.

Osteoclastogenesis is an indispensable step for the formation of the osteolytic lesion. Disseminated tumour cells can release multiple cytokines and growth factors to facilitate colonization. For example, RANKL and CSF-1, as the renowned osteoclastogenic factors, were liberated to promote the expression of hallmark genes of osteoclastogenesis including TRAP, CATK calcintonin receptor and β3-integrin, Jagged1, VCAM1, CTGF and IL11 leading to an activation of pre-osteoclast cells. PTHrP, IL-6 and IL-1 could stimulate bone resident osteoblasts to produce more cytokines, TNF-receptor-associated factor 6 (TRAF6), NF-KB, MAPK, and activator protein 1 (AP-1) act as pivotal mediators of RANL/RANKL signalling in osteoclastogenesis. These osteolytic cytokines contribute to the destruction of bone matrix and consequent release of osteolytic cytokines enriched in the bone matrix such as IL-6, IL-11, PTHrP and Jagged 1, thereby, leading to advanced bone destruction which is referred as a vicious cycle of bone metastasis [44]. In the present study, FAU (Finkel-Biskis-Reilly murine sarcoma virus (FBR-MuSV)-associated ubiquitously expressed gene), HHIPL1, RPP14, MCM6, RREB1 were the top five upregulated genes associated with osteoclastogenesis, whilst PSRC1 (Proline and Serine Rich Coiled-Coil 1), ELFN2 (Extracellular Leucine Rich Repeat and Fibronectin Type III Domain Containing 2) and MMAA (Metabolism of Cobalamin Associated A) were the top three downregulated genes that may be negatively associated with osteoclastogenesis (Fig. 9). FAU belongs to the ubiquitin family with little reported on its function. FAU was found to regulate platinum-resistance in ovarian cancer [45]. FAU levels were decreased in prostate cancer and lead to decreased cytotoxicity of UVC (ultraviolet C) via modulating Bcl-G apoptosis pathway [46]. FAU expression was positively correlated with DKK1, SOST (Sclerostin) and IL-1B. IL-1B can activate osteoclasts [47]. Loss- of-function of SOST was associated with sclerotises that is caused by progressive bone overgrowth [48]. Serum level of DKK1 is also associated with osteoporosis [49]. Taken together, FAU may act as a promising molecule in regulating the activity of osteoclasts to achieve osteoclastogenesis. HHIPL was able to enhance the hedgehog signalling activity to promote the atherosclerosis [50]. Hedgehog signalling also exerted an important role in bone development [51]. HHIPL was positively correlated with RANKL and IL6 in the current analyses, which indicated that HHIPL presented a potential to regulate osteoclastogenesis by co-acting with RANKL and IL to promote the maturation of the osteoclast via hedgehog signalling. Upregulated PSRC1 contributed to reduce the atherosclerosis by regulating the cholesterol transportation in apoE-/- mice [52]. ELFN2 is a vital postsynaptic adhesion molecule in brain behaviour [53] and ELFN2 signalling pathway also acts as the downstream molecule of CST4, which promoted aggressiveness in gastric cancer [54]. Little study has focused on the role of MMAA in bone metabolism. From the current analyses, PSRC1, ELFN2 and MMAA expression were inversely correlated with DKK1 and SOST in the current dataset. DKK1 and SOST were both negative bone formation regulators, which indicated that the three genes listed above may be involved in suppressing the role of osteoblasts to promote the osteoclastogenesis. Further mechanism underlying the osteoclastogenesis regulated by these genes and their exact implication in bone metastasis of breast cancer are yet to be investigated.

Although bone metastases for breast cancer are mainly osteolytic lesions, there are also osteogenic characteristics for breast cancer [55]. There are approximate 12–50% of breast cancers with bone metastases containing osteoblastic lesions [56]. In addition, bone destruction in osteolytic lesions can cause secondary new bone formation, leading to osteogenic changes [57], [58], which explains the existence of mixed lesions in breast cancer bone metastases. Recent research also revealed that bone marrow endothelium was capable of regulating the bone remodelling nearby [59]. Type H vessel, as an important subset of endothelia with high expression of CD31 and endomucin (EMCN), was shown to co-act with osteoblasts to promote the formation of trabecular bone. From the current analyses, RPP14, FAM117B, PPAT (Phosphoribosyl Pyrophosphate Amidotransferase), ARMC2 (Armadillo Repeat Containing 2), SNX27 (Sorting Nexin 27) and ZZZ3 presented high correlation with the osteoblastic determining genes (Fig. 9). RPP14 is a kind of eukaryotic endoribonuclease preferentially correlated with nucleolar and mitochondrial RNA processing [60]. No previous report was found on the impact of RPP14 in bone metabolism or breast cancer. From the current analyses, RPP14 is closely correlated with BMP4, BMP7 and EDN1 in the bone metastatic lesion, which reflects the potential role of RPP14 in bone formation. Little research was performed on FAM117B. According to Fischer's study, FAM117B was identified as the immune related factors associated with sarcoidosis in European populations [61]. FAM117B is highly correlated with BMP4 and BMP7 in the current analyses, which indicates the potential role of FAM117B in facilitating the bone formation. Research of PPAT in lung adenocarcinomas showed that, PPAT regulated tumour progression via purine biosynthetic pathway [62]. PPAT was also known to be involved in the amino acid metabolism and mutate in gastrointestinal tumours [63]. ARMC2 was found to be involved in male infertility caused by the multiple morphological abnormalities of the sperm flagella (MMAF) [64]. PPAT presented positive association with PDGFA (Platelet Derived Growth Factor Subunit A) and WNT10B (Wingless-Type MMTV Integration Site Family, Member 10B) in the present analyses, whilst ARMC2 was negatively correlated with osteoblastic regulators BMP4, BMP7 and EDN1, which indicated the potential role of PPAT and ARMC2 in the osteoblastogenesis. Further investigation of the osteoblastic activities in bone metastases from breast cancer will help us to provide a more balanced and tailored treatment.

Importance of bone marrow in haematopoiesis confers itself a pivotal role in systemic immunity by producing a couple of immune cells such as cytotoxic T, natural killer (NK) cells and regulatory T cells (Treg) and myeloid derived suppressor cells (MDSC) [65]. The immune system is closely correlated with the progressive colonisation of tumour in bone. The immune system and bone share many common signal transduction pathways, Treg cells, as the immune suppresser, were induced to the bone marrow via CXCR4/CXCL12 signalling [66]. Osteoblasts can regulate the proliferation of hematopoietic cells and the differentiation of B cells [67], whilst Osteoclasts are derived from monocyte precursor cells, and macrophages and monocytes are also differentiated from monocyte precursor cells. RANKL signalling exerts pivotal role in the development of immune organs, which was previously reported to be the activator of dendritic cells expressed by T cells [68]. From the current analyses, FAS, C16orfS4, FBX031, ACSS3, ZCCHC3 and THBS3 were identified as the promising genes associated with immune escape (Fig. 9). FAS, also known as CD95, belongs to the tumour necrosis factor receptor (TNFR) superfamily. Previous study showed that FAS mutation is correlated with attenuated apoptotic signalling, research in recent years also revealed that FAS is involved in the nonapoptotic signals which promoted oncogenesis [69]. FAS-mediated apoptosis may have a role in the induction of peripheral tolerance and/or antigen-stimulated suicide of mature T-cells. FAS expression was positively associated with CD18, CD47 and IRF47, which suggested that FAS may also help the migrated tumour cells survive from the immune surveillance. As one of the muscle transcripts, ACSS3 was applied as the marker to determine the bone mineral density (BMD). ACSS3 was also found to downregulated and associated with poor prognosis in prostate cancer [70]. Whilst upregulated ACSS3 expression in gastric cancer was correlated with the tumour proliferation and invasion [71]. Expression of ACSS3 was inversely associated with ITGB2 and IRF7 in the present research. ITGB2 plays a vital role in immune response, silencing ITGB2 leads to leukocyte adhesion deficiency and IRF7 is associated with immunodeficiency, enhanced expression of ACSS3 will help the migrated cells to evade from the immune elimination.

Tumour associated angiogenesis (also known as neovascularisation) is a crucial hallmark of cancer. Bone marrow endothelium is also involved in angiogenesis in the bone lesions, in which vascular endothelial growth factor (VEGF), hypoxia-inducible factor 1a (HIF1A) and PTHrP play a profound role [72], [73]. Additionally, the bone marrow endothelium was identified to promote the maturation of osteoblasts and depletion of osterix (OSX) in endothelial cells suppress the formation of osteoblastic lesion [74]. From the current analyses, upregulated ZNF519 (Zinc Finger Protein 519), UTRN, CSPG4 and downregulated ALDHA2, GLRA1 and DPYS were top promising genes associated with angiogenesis in the bone lesion (Fig. 9). CSPG4 is a surface type I transmembrane core proteoglycan, which is actively involved in the cell progression including angiogenesis [75]. In a previous study, CSPG4 was found to induce abnormal uterine bleeding via endometrial angiogenesis [76]. In the current analyses, CSPG4 was positively associated with CD34 and HIF1A, together with the previous report on the role of CSPG4 in angiogenesis, CSPG4 tends to be a promising molecule to be evaluated for its involvement in bone metastasis related angiogenesis. Aldehyde dehydrogenases (ALDHs) were found to be highly-expressed in various tumours [77] which regulate the proliferation of tumour cells [78]. ALDH inhibitors also presented anticancer potential [77]. In the present study, we found that ALDHA2 expression was inversely correlated with CD34 and S1PR1. The anti-angiogenic effect on the bone metastasis associated angiogenesis by targeting ALDHA2 provokes further investigation.

Regional irradiation is beneficial for the pain control and skeletal integrity maintenance, although it can cause a greater risk of bone fraction [79]. Irradiation-induced bone matrix dissolution can be seen 1 week after the irradiation exposure, in which altered collagen crosslinking was involved [80]. In addition to the local radiotherapy, target therapy also presents promising effect in the treatment of bone metastases. Currently, target drugs are designed mainly to regulate the activity of osteoclast and osteoblast. Bisphosphonates, a widely used inhibitor of bone resorption, is capable of attenuating the mevalonate pathway, which is essential for the osteoclast activation [81]. Denosumab works as the anti-RANKL monoclonal antibody to suppress the differentiation and activity of osteoclast [81]. c-Src inhibitors can also target on the RANKL induced osteoclast differentiation [82]. Mammalian target of Rapamycin inhibitors are used to restrain the osteoclast differentiation and promote osteoclast apoptosis [81]. Targeting Wnt pathway may be helpful to rescue osteoblastic activity in the bone metastatic lesion [83]. Comparing to the rapid development of new therapeutic approaches, our knowledge about histology and gene expression in bone metastases from patients who received target therapy or radiotherapy remains poor as lack of specimens, re-examination of histology and corresponding genomic and proteomic analysis.

## 5 Conclusions

Bone metastasis is an invasive, sequential, dynamic, adaptive and interactive process. Nine genes were identified as promising genes associated with the predisposition of bone metastasis. GDF11 was screened as the most promising gene associated with the homing, osteoclastogenesis and osteoblastogenesis in the bone metastasis of the breast cancer. CD151 and PAFAH1B2 may actively participate in the angiogenesis of bone lesion. When disseminated cancer cell migrated to the bone, a series of adaptive phenotypes including colonization, immune escape, angiogenesis, osteoblastogenesis and osteoclastogenesis were experienced to form the final bone metastasis. According to the current analyses, DPP9, LRRC20, TTBK2, TTC17, ZNF75D and CYTH2 were highly correlated with the homing of the migrated cell. FAS, C16orfS4, FBXO31, ACSS3, ZCCHC8 and THBS3 were found to help the metastatic cancer cells survive from the immune surveillance. ZNF519, UTRN, CSPG4, ALDHA2, GLRA1 and DPYS may play a profound role in the bone lesion associated angiogenesis to facilitate the progress of bone metastasis. RPP14, FAM117B, PPAT, ARMC2, SNX27 and ZZZ3 may help to activate the osteoblasts to form the osteoblastic bone metastasis, whilst FAU, HHIPL1, RPP14, PSRC1, ELFN2 and MMAA may exert potential role in the osteoclastogenesis. Bone metastasis is a complex procedure regulated by groups of molecules, exact roles of the identified genes in the bone metastasis still needs to be further explored.

**Authors contribution**.

LY and WGJ designed the study. LS, AS and LY performed data analysis and statistical analysis. LS, AS, WGJ and LY drafted and revised the manuscript.

## Declaration of Competing Interest

The authors declare that they have no known competing financial interests or personal relationships that could have appeared to influence the work reported in this paper.
